# Solution-Based Single-Molecule FRET Studies of K^+^ Channel Gating in a Lipid Bilayer

**DOI:** 10.1016/j.bpj.2016.05.020

**Published:** 2016-06-21

**Authors:** Emma E. Sadler, Achillefs N. Kapanidis, Stephen J. Tucker

**Affiliations:** 1 Clarendon Laboratory, Department of Physics, University of Oxford, Oxford, United Kingdom

## Abstract

Ion channels are dynamic multimeric proteins that often undergo multiple unsynchronized structural movements as they switch between their open and closed states. Such structural changes are difficult to measure within the context of a native lipid bilayer and have often been monitored via macroscopic changes in Förster resonance energy transfer (FRET) between probes attached to different parts of the protein. However, the resolution of this approach is limited by ensemble averaging of structurally heterogeneous subpopulations. These problems can be overcome by measurement of FRET in single molecules, but this presents many challenges, in particular the ability to control labeling of subunits within a multimeric protein with acceptor and donor fluorophores, as well as the requirement to image large numbers of individual molecules in a membrane environment. To address these challenges, we randomly labeled tetrameric KirBac1.1 potassium channels, reconstituted them into lipid nanodiscs, and performed single-molecule FRET confocal microscopy with alternating-laser excitation as the channels diffused in solution. These solution-based single-molecule FRET measurements of a multimeric ion channel in a lipid bilayer have allowed us to probe the structural changes that occur upon channel activation and inhibition. Our results provide direct evidence of the twist-to-shrink movement of the helix bundle crossing during channel gating and demonstrate how this method might be applied to real-time structural studies of ion channel gating.

## Introduction

Ion channels are complex multimeric structures, and understanding how they open and close represents a major technical challenge (1, 2, 3). The study of ion channel structures by x-ray crystallography has made many significant advances in this field, but the functional landscape of a channel explores multiple structural states, many of which are not easily trapped in crystallographic forms. Channel gating is also stochastic and inhomogeneous, and often involves unsynchronized structural transitions (2, 3, 4). Furthermore, the relative stability of these different states can be highly dependent upon the lipid environment, which is often disturbed upon detergent solubilization; for example, once isolated from the membrane, some channels preferentially crystallize in the closed state and have to be forced into the open state by mutagenesis (4, 5, 6). These problems are in marked contrast to functional studies of ion channels, which for many decades have employed electrophysiological methods to obtain high-resolution data from single ion channels in a native membrane environment (7). Better methods for dynamic structural studies of single ion channels are therefore required to fully complement these detailed functional approaches.

To address this challenge, we reconstituted KirBac1.1, a prokaryotic homolog of an inwardly rectifying (Kir) potassium channel, into lipid nanodiscs and performed single-molecule fluorescence studies of channel gating using Förster resonance energy transfer (FRET) with confocal-in-solution alternating-laser excitation (ALEX) microscopy. This technique examines fluorescently labeled molecules traversing a femtoliter observation volume and uses ALEX to assign fluorescence events to a specific subpopulation (see Fig. S1 in the Supporting Material for a detailed description). This method is routinely used to study conformational changes in monomeric, soluble proteins (8, 9, 10, 11) (see also Fig. S1, *B* and *C*). The ability of ALEX to accurately differentiate FRET signals from the donor and acceptor fluorophores is vital for distinguishing subpopulations and hence for examining dynamic heterogeneity within a sample that has several states existing in a dynamic equilibrium sensitive to outside perturbation. Such heterogeneity is often obscured by macroscopic measurements that generate an ensemble-averaged estimation of a single intermediate population that may not actually exist (11).

Single-molecule FRET (smFRET) is highly efficient at reporting changes in inter- and intramolecular distances, such as those that occur during ion channel gating. It also provides a very effective way to examine the different populations that exist in equilibrium between two or more structural states, which is inaccessible to most ensemble measurements, especially for a multimeric protein. Furthermore, although bulk solution measurements can demonstrate the presence of two conformational states with a constant fraction of proteins in each state, the lack of temporal and molecular resolution means that one cannot determine whether individual proteins remain stable in a particular state (static heterogeneity) or each molecule is switching between two states in a dynamic equilibrium (dynamic heterogeneity).

Some early smFRET studies of membrane proteins examined the association of gramicidin (12), while more recent attempts have studied the conformational dynamics of the Na^+^-coupled aspartate transporter GltPh (13, 14) and the mechanosensitive ion channel MscL (15). However, GltPh is a monomeric protein, and in both experimental systems the proteins require tethering, either directly or via reconstitution into a liposome, to a coverslip using a biotin-avidin linker, and the resulting signals are then measured using total internal reflection fluorescence (TIRF) microscopy.

The approach we describe here exploits the use of nanodiscs to solubilize the channels, thereby allowing ALEX confocal-in-solution microscopy to be combined with smFRET (8, 9). Furthermore, the problem of using a multimeric protein where more than one labeling site exists is solved by using substoichiometric fluorescent labeling to produce populations of channels with both donor and acceptor fluorophores attached. These randomly labeled populations of channels are then examined as they traverse a femtoliter observation volume. Dilution of the sample means that only one channel is likely to be diffusing through the observation volume at any one time, and the characteristics of the four distinct photon streams resulting from ALEX can be used to assign fluorescence events to a particular subpopulation based on their FRET efficiency (*E^∗^*) and label stoichiometry (*S*) (see Supporting Materials and Methods for details).

This sorting method, known as fluorescence-aided molecular sorting (FAMS) (8, 9), allows population histograms to be constructed and multiple labels to be distinguished within a multimeric protein (e.g., a homotetrameric K^+^ channel) where the use of a single cysteine reporter mutation produces four possible sites at which donor and/or acceptor fluorophores can attach (see also Fig. S1). The approach therefore does not require concatenation of subunits to restrict the number of sites that might get labeled, which has previously presented a major obstacle for the application of smFRET to multimeric ion channels.

Solution-based smFRET also has several advantages over alternative methods that require wide-field imaging and/or tracking of individual molecules, but such an approach has never previously been applied to study conformational changes in a membrane protein. This is primarily due to the obvious difficulties associated with solubilization of membrane proteins. However, nanodiscs provide membrane proteins with a more native bilayer-like environment that confers a high degree of stability as well as a resistance to protein aggregation and reduced autofluorescence (16, 17, 18). Nanodiscs are also monodisperse and the lipid composition can be tailored to meet the requirements of the target protein (19, 20). Furthermore, many different membrane proteins appear to retain their functional properties in nanodiscs (19, 20, 21, 22, 23).

Using this single-molecule approach, we observed fluorescence changes in subpopulations of KirBac1.1 in response to changes in pH, PIP_2_ (2, 24, 25, 26, 27), and an activatory mutation (25), all of which are known to affect the equilibrium between the open and closed states of the channel. The results demonstrate how this solution-based single-molecule technique can now be applied to study the gating of multimeric membrane proteins in a bilayer-like environment.

## Materials and Methods

### Molecular biology and protein expression

A codon-optimized version of the KirBac1.1 open reading frame was custom synthesized (Genscript, Piscataway, NJ) to reduce its guanine-cytosine content and subcloned into pQE60Lac using NcoI and *Hin*dIII cleavage sites for expression in BL2121-Gold(DE3) pLysS Competent Cells (Agilent UK, Stockport, UK). Oligonucleotide-based, site-directed mutagenesis was then used to introduce point mutations. For expression, 50 mL LB starter cultures were inoculated and grown overnight at 37°C and 210 rpm. Then, 15 mL/L of these cultures was used to inoculate LB in baffled flasks that were incubated at 37°C and 190 rpm until OD_600_ = 1.1. Expression was induced using 1 mM isopropyl-*β*-D-1-thiogalactopyranoside (Sigma Aldrich, St. Louis, MO) and cultures were incubated overnight at 19°C and 190 rpm.

### KirBac1.1 purification

Pellets were harvested at 16,000 *g*, resuspended in 12.5 mL buffer per liter of original culture (50 mM Tris-HCl pH 7.8, 150 mM KCl, 10 mM MgCl_2_, 250 mM sucrose, 5 mM *β*-mercaptoethanol, one tablet/50 mL EDTA-free protease inhibitor (Roche, Burgess Hill, UK), 10 mg/mL hen egg white lysozyme (Fluka, Gillingham, UK), and 10 mg/mL DNaseI (Sigma)). Cells were lysed by six to eight passes through a TC5 homogenizer (Stansted Fluid Power, Harlow, UK). n-Dodecyl-*β*-D-maltoside (DM; Anatrace, Maumee, OH) was added to a final concentration of 30 mM and the mixture was solubilized at 4°C for 1–2 h. Solubilized lysate was centrifuged (70,000 *g* for 40 min) to extract cell debris, and the supernatant was added to Amintra CoHIS resin (Expedeon, Swavesey, UK) that had been equilibrated with 20 volumes of buffer (50 mM Tris-HCl pH 7.8, 150 mM KCl) and incubated at 4°C for 1–2 h for affinity purification. Unbound protein was collected and the resin was washed with 20 volumes of buffer (50 mM Tris-HCl pH 7.8, 150 mM KCl, 5 mM DM, 10 mM imidazole) and eluted with 10 volumes of buffer (50 mM Tris-HCl pH 7.8, 150 mM KCl, 5 mM DM, 500 mM imidazole) after a 20 min incubation. Concentrated eluate was loaded onto a size-exclusion column (Superdex 200 10/300 (GE Healthcare, Little Chalfont, UK) equilibrated with 50 mM Tris-HCl pH 7.8, 150 mM KCl, 5 mM DM) to separate and collect the tetrameric fraction.

### KirBac1.1 labeling

To randomly incorporate both donor and acceptor fluorophores into the channel, substoichiometric labeling with a mixture corresponding to protein/donor/acceptor ratios of 10^4^:8:4 was performed. A reaction mixture of 200 *μ*L KirBac1.1, 1 *μ*L Cy3B maleimide (GE Healthcare), and 0.5 *μ*L Alexa Fluor 647 maleimide (Invitrogen, Carlsbad, CA) was made and incubated in the dark at room temperature for 1 h. It was then added to Amintra CoHIS resin (equilibrated with 20 volumes of 20 mM HEPES pH 7.5, 150 mM KCl) and incubated at 4°C for 1 h. Affinity purification was performed by washing with 20 volumes of wash buffer (20 mM HEPES pH 7.5, 150 mM KCl, 5 mM DM, 10 mM imidazole) followed by elution with 10 volumes of elution buffer (20 mM HEPES pH 7.5, 150 mM KCl, 5 mM DM, 500 mM imidazole) after a 15 min incubation. The eluate was run through an equilibrated NAP-5 column (GE Healthcare) and six 0.5 mL fractions were collected. Wild-type KirBac1.1 contains no endogenous cysteines, and consistent with previous reports (26), no background labeling was observed. The labeling efficiency of reporter cysteine mutants was estimated by fluorescent visualization of proteins on an SDS-PAGE gel (Nu-PAGE 4-12% Bis-Tris; Novex, Waltham, MA) and visualized using a Pharos FXTM molecular imager (BioRad, Hercules, CA) with Discovery Series Quantity One v4.6.9 software to determine labeled fractions.

### Membrane scaffold protein expression and purification

For generation of nanodiscs, the MSP1E3D1 gene was subcloned into the pET28a vector and transformed into BL21-Gold (DE3) pLysS Competent Cells. A 15 mL overnight starter culture was used to inoculate 2 L of Terrific Broth (Sigma Aldrich) that was incubated at 37°C and 190 rpm until OD_600_ > 1.4, when expression was induced with 1 mM isopropyl-*β*-D-1-thiogalactopyranoside and cultures were incubated for another 3 h. Cells were harvested by 15 min centrifugation at 16,000 *g* and resuspended in 50 mL phosphate-buffered saline (Sigma) containing 1% Triton X-100, 10 mg/mL hen egg white lysozyme, one cocktail tablet/50 mL EDTA-free protease inhibitor, and 10 mg/mL DNaseI. They were then lysed and centrifuged at 70,000 *g* for 30 min. Supernatant was added to washed and equilibrated Amintra CoHIS resin (buffer: 40 mM Tris-HCl pH 8.0, 300 mM NaCl) and incubated at 4°C for 1 h. Unbound protein was collected, the resin was washed with 20 volumes of W1 buffer (40 mM Tris-HCl pH 8.0, 300 mM NaCl, 1% Triton X-100) and 20 volumes of W2 buffer (40 mM Tris-HCl pH 8.0, 300 mM NaCl), and was elution performed with 10 volumes of 40 mM Tris-HCl pH 8.0, 300 mM NaCl, 500 mM imidazole. Buffer exchange of the eluted fraction to 20 mM Tris-HCl pH 7.8, 100 mM NaCl, 0.5 mM EDTA was also performed. Concentrated eluate was visualized using SDS-PAGE.

### Nanodisc assembly

Reagents were mixed to obtain a total volume of 800 *μ*L, including KirBac/membrane scaffold protein (KirBac/MSP) in a 1:1 molar ratio: 200 *μ*L KirBac1.1 (2 mg/mL); 100 *μ*L MSP (2 mg/mL), 100 *μ*L POPE (10 mM); 34 *μ*L POPG (10 mM), 166 *μ*L cholate buffer (20 mM Tris-HCl pH 7.8, 100 mM NaCl, 0.5 mM EDTA, 100 mM cholate); and 200 *μ*L of plain buffer (20 mM Tris-HCl pH 7.8, 100 mM NaCl, 0.5 mM EDTA). The reaction mixture was incubated overnight at 4°C with gentle agitation, added to BioBeads equilibrated with plain buffer, and incubated for 6–8 h before it was loaded onto a size-exclusion column (Superdex 200 10/300) for purification.

### Single-molecule confocal experiments

Double-labeled KirBac1.1 was present at 5–20 nM in the observation buffer (20 mM HEPES pH 7.5, 150 mM KCl, 0.5 mM EDTA). For experiments examining inhibition by PIP_2_, 20 *μ*M diC8-PIP_2_ was added to the observation buffer. For experiments examining inhibition by acidic pH, the observation buffer contained 50 mM 2-(N-morpholino)ethanesulfonic acid, pH 5.5, as a substitute for the HEPES (20 mM, pH 7.5) control. For measurements of relative shifts upon activation/inhibition, all samples were compared with controls calibrated on the same day. Experiments were performed at room temperature (18–22°C) using a confocal microscope with ALEX at 10 kHz between 532 nm (120 *μ*W continuous wave; Samba, Cobalt, Solna, Sweden) and 635 nm (30 *μ*W continuous wave; CUBE, Coherent, Santa Clara, CA) as described previously (10) and illustrated in Fig. S1. Aqueous samples were pipetted directly onto a coverslip resting on top of an oil immersion objective (60×, NA = 1.35, UPLSA 60XO; Olympus, Southend-on-Sea, UK) focused to 20 *μ*m above the coverslip surface, and observed for 600–900 s with a frame rate of 1 ms, assuming a single-labeled channel would diffuse across the femtoliter observation volume in ∼10 ms.

### Data analysis

Photon arrival times in each channel were recorded by separate avalanche photodiodes (SPCM-AQR-14; PerkinElmer, Beaconsfield, UK) and analyzed using custom-written software in LabVIEW (National Instruments, Newbury, UK) and MATLAB (The MathWorks, Natick, MA). Photon bursts were identified using previously described algorithms (8, 9) that acknowledge photons arriving at the avalanche-photodiodes accompanied by a threshold number of neighboring photons arriving within a threshold time interval, and a filter was applied such that only bursts with an activated acceptor fluorophore were examined (see Supporting Materials and Methods for further mathematical description). *E^∗^* and *S* were collated into two-dimensional histograms and peak positions were obtained by fitting to double-Gaussian functions. Additionally, the standard deviation (SD) of *E^∗^* of each burst was calculated using a sliding window of 20 photons along the donor excitation stream. This was compared with the shot noise limit, and an SD greater than the shot noise was determined to be indicative of protein dynamics rather than an intrinsic property of the avalanche-photodiodes.

The smFRET values presented in the figures are uncorrected values. Cross talk between donor and acceptor channels, differences in the efficiency of fluorescence transfer and collection, and differences in quantum yield between the donor and acceptor can account for significant discrepancies in absolute versus raw values, but after correction, relative shifts in *E^∗^* remain unchanged (see Supporting Materials and Methods for further details).

## Results

### Validation of the methodology

**Figure 1:**
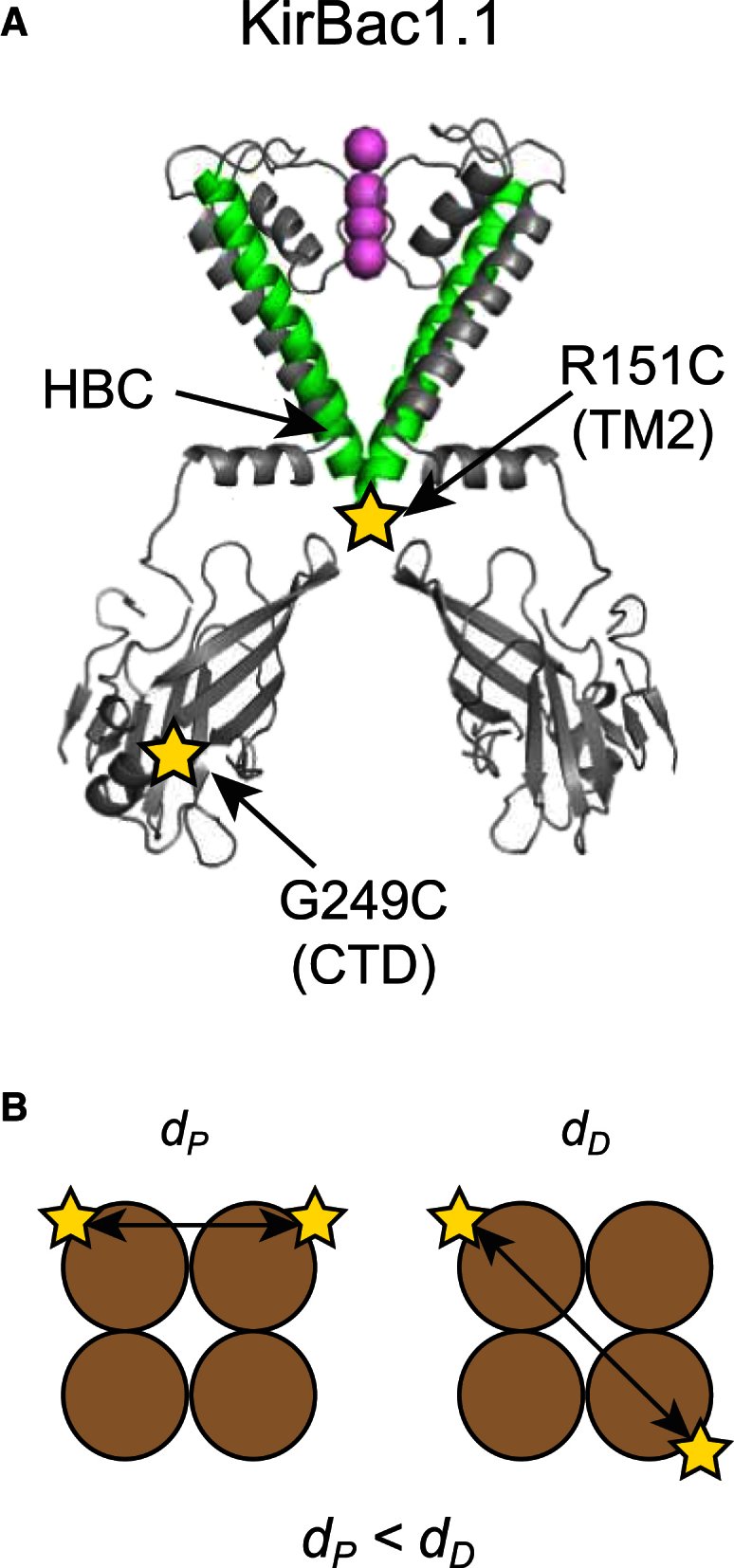
(*A*) The relative locations of the two separate cysteine mutation reporter sites used in this study are shown; R151C is located at the base of TM2 and is below the HBC gate. G249C is in the CTD. K^+^ ions within the filter are shown as spheres, but, for clarity, only two of the four identical KirBac1.1 subunits are shown. (*B*) Due to the multimeric nature of the channel, even when the channel is labeled by only single donor and acceptor fluorophores, there are still two possible labeling schemes in which the interdye distances are different. The relative proximal (*d*_*P*_) and distal (*d*_*D*_) distances are shown. Note that *d*_*P*_*< d*_*D*_. To see this figure in color, go online.

KirBac1.1 was expressed and purified by adaptation of established protocols (see Materials and Methods for details). Initial attempts to record smFRET using detergent-solubilized protein were marred by protein aggregation and degradation, and did not produce distinct fluorescence populations. Therefore, the channel protein was placed into a more native-bilayer-like environment via reconstitution into nanodiscs (MSP1E3D1; see Materials and Methods for details). Before reconstitution, KirBac1.1 was labeled with both donor (Cy3B) and acceptor (Alexa Fluor 647) fluorophores via cysteine-maleimide labeling at substoichiometric ratios. Since wild-type KirBac1.1 has no endogenous cysteine residues, single-labeling sites were introduced into either the second transmembrane (TM) helix (R151C in TM2) just below the helix bundle crossing (HBC) or at a position (G249C) in the C-terminal domain (CTD) that is well separated from the membrane-embedded region (Fig. 1). Both of these sites were previously used to monitor macroscopic changes in FRET during KirBac1.1 gating and the mutants retained normal functional activity (27).

However, KirBac1.1 is a homotetramer with four potential cysteine-maleimide labeling sites per channel, and labeling will occur randomly, especially when performed substoichiometrically (protein/dye ratio > 1000:1). Thus, even with a single reporter site, it becomes possible to label either two adjacent subunits (proximal) or those diagonally opposite to each other (distal) as shown in Fig. 1. FAMS can distinguish between these two populations by their *E^∗^* values because the interdye distances will differ. An offset in *S* also often occurs, but this results from differences in the physical properties of the donor/acceptor such as the quantum yield and excitation efficiency. Importantly, this method also excludes any donor/acceptor-only and D-D/A-A events. Both the proximal and distal fluorescent populations were monitored in this study because both distances should undergo similar changes during channel gating (i.e., an increase or decrease in distance). The ability to distinguish similar changes in two close populations acts as a useful control.

**Figure 2:**
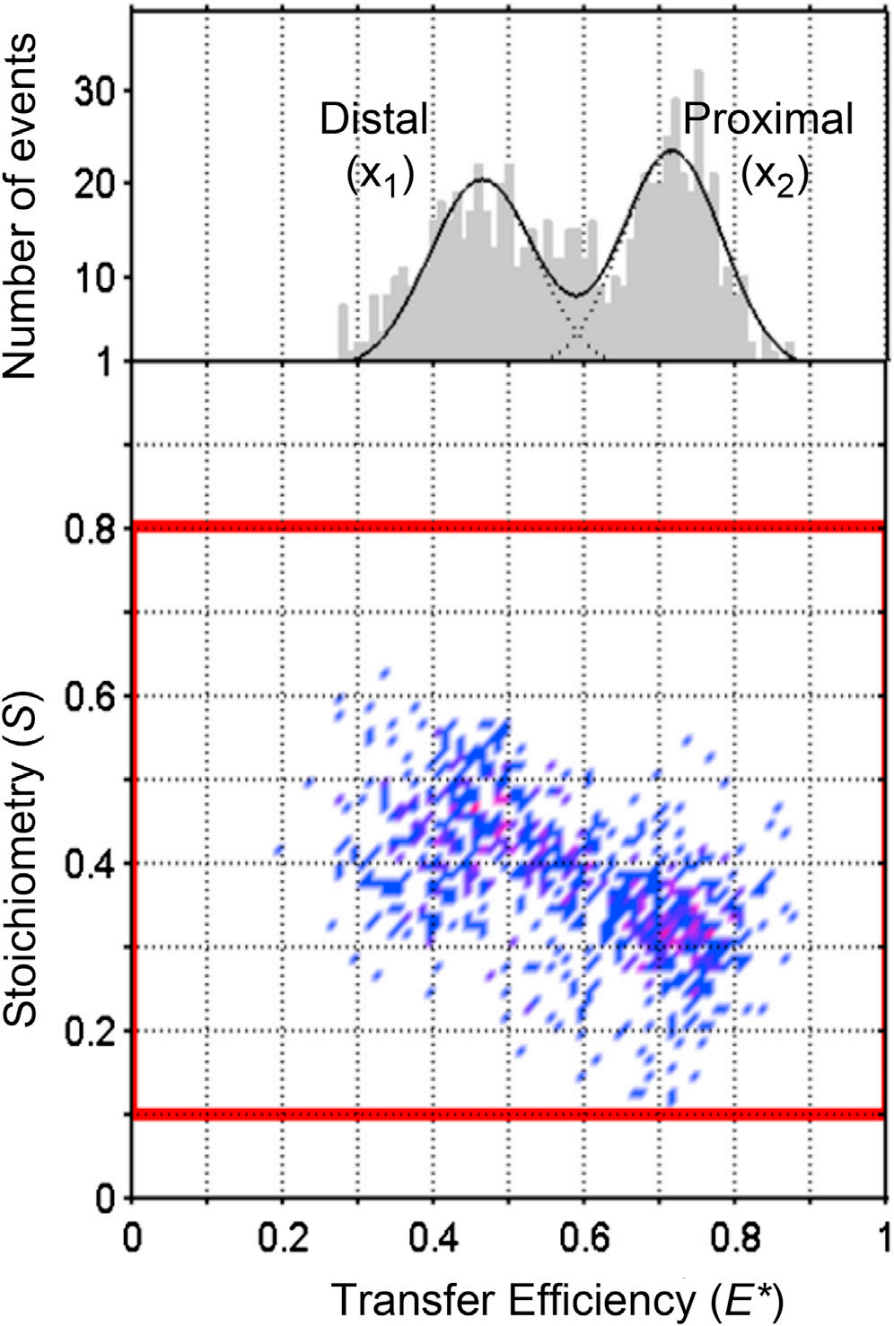
*E^∗^-S* histogram obtained during an smFRET study of KirBac1.1; the example shown is for the R151C mutant. The two populations (X_1_ and X_2_) refer to the proximal and distal distances (*d*_*P*_ and *d*_*D*_), which are different (*d*_*P*_*< d*_*D*_), and hence the transfer efficiency is lower for the distal (X_1_) population than for the proximal (X_2_) population. An offset in *S* occurs due to differences in the physical properties of the donor and acceptor fluorophores. The red box indicates the collapsed FRET data for the donor/acceptor species only. Other species occur (Fig. S1), but with *S* > 0.9 or *S* < 0.2. To see this figure in color, go online.

After initial optimization of the labeling efficiency, confocal-in-solution smFRET experiments with double-labeled KirBac1.1 allowed resolution of both proximal and distal fluorescence populations. An example of collapsed FRET data (i.e., donor/acceptor-only species) for the R151C reporter site is presented in Fig. 2, which shows two populations for the proximal and distal distances (*d*_*P*_ and *d*_*D*_). Other species occurred, but these were far away from the donor/acceptor species and thus are not shown in the figure (8). To verify the identity of these populations, we used previously characterized methods of activating and inhibiting channel activity, with the aim of shifting the open-closed state equilibrium of the channel and hence producing a shift in *E^∗^* in a predictable manner. An activatory gain-of-function mutation (V145L) has been shown to increase the open probability of KirBac1.1, i.e., to shift the dynamic equilibrium toward the open state (25). Likewise, the gating equilibrium can also be shifted in favor of the closed state by inhibiting KirBac1.1 with acidic pH or exposing it to PIP_2_ (which inhibits prokaryotic KirBac channels) (2, 24).

**Figure 3:**
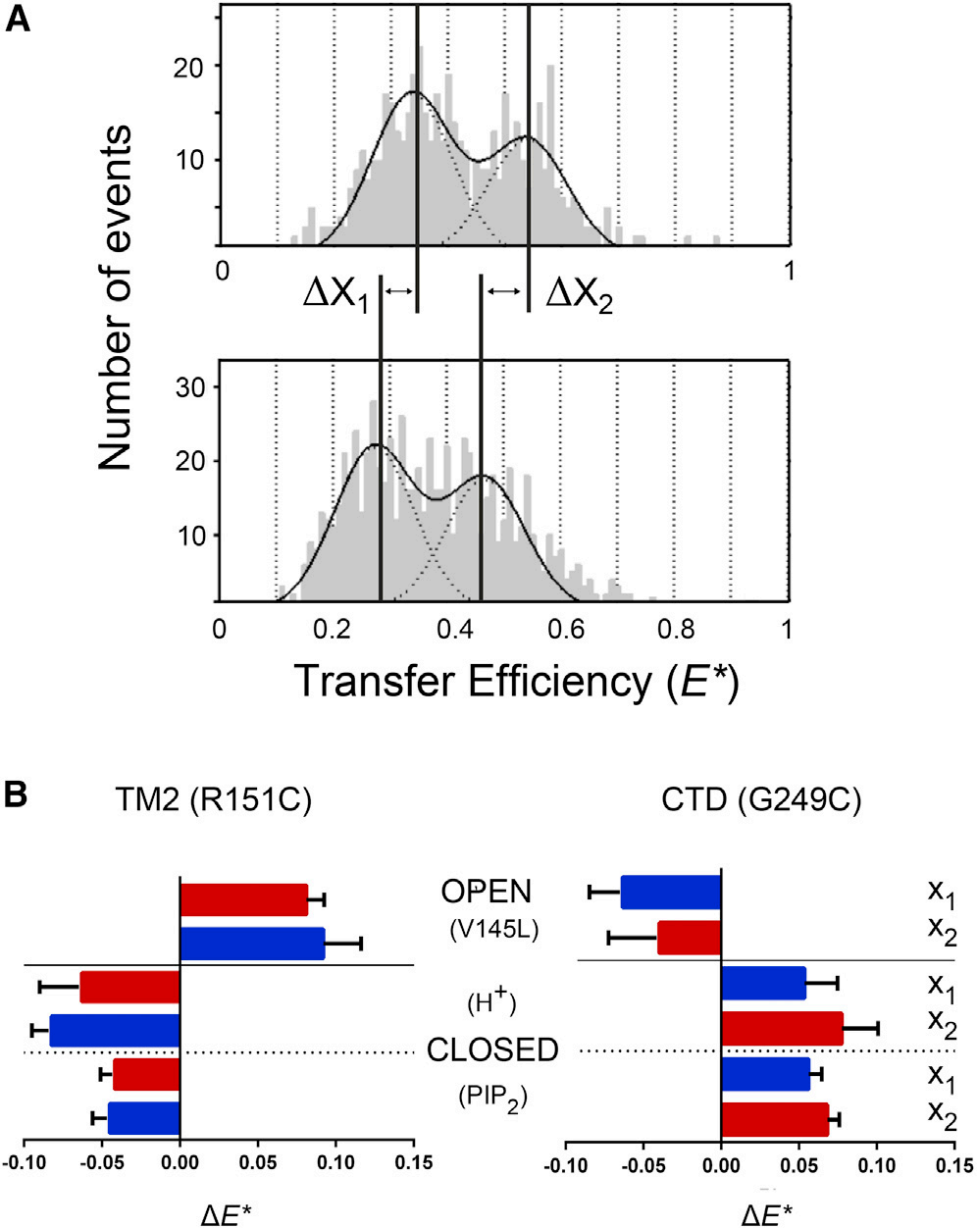
(*A*) Example *E^∗^* histograms showing the shifts in *E^∗^* upon intracellular acidification for the R151C reporter site. It can be seen that the distal and proximal distances were shifted to lower values of *E^∗^* upon channel inhibition by H^+^. (*B*) Summary of the shifts in smFRET efficiency observed upon a change in the open-/closed-state gating equilibrium via three different mechanisms. Importantly, the shifts for the two reporter sites in TM2 and the CTD are in opposite directions. Also, channel activation using the V145L activatory mutation produces a shift in the opposite direction to channel inhibition (using either H^+^ or PIP_2_). The data shown refer to the mean ± SD. X_1_ and X_2_ refer to the distal and proximal peaks as shown in Fig. 1. To see this figure in color, go online.

These known regulatory mechanisms all produced shifts in *E^∗^*, as measured from the centers of the Gaussian fits in the *E^∗^-S* histograms, indicating that conformational changes occurred near the two different reporter sites. This is illustrated in Fig. 3 *A* for the R151C reporter site. The shifts for both proximal and distal distances are summarized in Fig. 3 *B*. The distance changes taken from the histograms are relative, not absolute, because correction factors were not included; nevertheless, the distances can be calculated and agree with the expected distances calculated from closed-state crystal structures of KirBac1.1. Furthermore, the modeling of the positions of the probes and linkers when constrained by the protein surface is consistent with the results presented below (see Supporting Materials and Methods for details, including postcorrection values for FRET shifts).

### Changes in response to inhibition by acidic pH

As shown in Fig. 3, when KirBac1.1 was exposed to acidic pH, which inhibits channel activity, a shift (average magnitude ∼0.07) toward lower *E^∗^* was observed when the R151C reporter mutation was used, indicating an increase in the interdye distance near the HBC (see also Fig. S2). By contrast, a shift (average magnitude ∼0.065) toward a higher *E^∗^* was observed for the G249C reporter, indicating a decrease in distance at this location within the CTD. Importantly, there was no observed effect of pH changes on the free dyes themselves, or FRET between them when attached to fixed sites on a length of double-stranded DNA (10).

### Effect of inhibitory phosphoinositides

Although PIP_2_ produces a marked activation of eukaryotic Kir channels, this phosphoinositide is a well-characterized inhibitor of prokaryotic KirBac channels. Therefore, we examined whether this inhibitory mechanism produces structural changes in KirBac1.1 similar to those produced by acidic pH. As shown in Figs. 3 *B* and S2, a shift toward lower *E^∗^* (i.e., an increase in dye separation) was also observed upon addition of di-C8-PIP_2_ when the R151C reporter mutation was used, whereas a shift toward higher *E^∗^* (a decrease in separation) was observed for the G249C reporter site. These shifts were both in the same direction and of approximately the same magnitude as those observed with inhibitory pH, suggesting that similar structural changes occur in response to channel inhibition.

### Changes in response to channel activation

**Figure 4:**
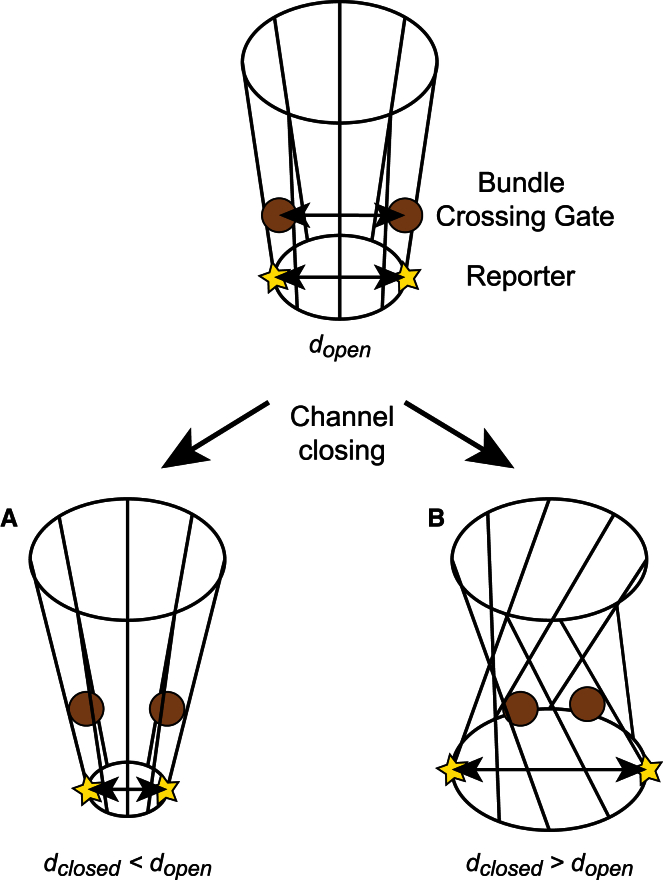
Geometric models for gating a K^+^ channel with an HBC gate. (*A* and *B*) The gate in an open channel (*top*) can close via simple dilation (*A*) or a twist-to-shrink mechanism (*B*). The residues that form the gate (F146 marking the bundle crossing) are represented by spheres, and the R151C smFRET reporter site is shown as a star. Note that the reporter site is located below the HBC gate. In model *A*, *d*_*open*_ > *d*_*closed*_, whereas in the twist-to-shrink model (*B*) this distance increases, i.e., *d*_*open*_ < *d*_*closed*_. The data are consistent with model *B*. To see this figure in color, go online.

Currently, there are no known regulatory mechanisms that can produce specific activation of KirBac channels. However, a number of activatory gain-of-function mutations have been identified. The V145L mutation in TM2 is one such mutation, and therefore we introduced it into TM2 and measured the changes in smFRET. Compared with the reporter-only variants of KirBac1.1, this activatory mutation produced a shift to higher *E^∗^* for the R151C reporter site and a shift to lower *E^∗^* for the G249C reporter mutation. These shifts were similar in magnitude to those observed upon channel inhibition, but were in the opposite direction for each reporter site, as shown in Fig. 3 *B* (see also Fig. S2). Overall, the geometry of movement of the R151C reporter site in TM2 relative to the position of the HBC gate at F146 provides strong support for the twist-to-shrink model of pore closure as shown in Fig. 4 (see the discussion below for more details).

## Discussion

In this study we have shown how confocal-in-solution ALEX microscopy combined with FAMS can be applied to measure structural changes in single molecules of a multimeric membrane protein, thereby removing the heterogeneity that comes with ensemble macroscopic measurements. This solution-based method also has advantages over other smFRET methods that require tracking of single molecules or wide-field imaging of surface-tethered molecules (26).

The two labeling sites reported FRET shifts (for both *d*_*P*_ and *d*_*D*_) in opposite directions, indicating opposing changes in these distances during channel gating, e.g., both inhibitory H^+^ and PIP2 produced a decrease in site separation at R151C, but an increase at G249C. These two reporter sites are positioned in different structural domains, and several studies have suggested that these domains undergo different twisting and tilting motions during channel gating (3, 4). Importantly, both inhibitory ligands produced shifts in the same direction and of similar magnitude for the two reporter sites, suggesting a common conformational change upon channel inhibition. Furthermore, the V145L activatory mutation produced changes of opposite but equal magnitude in *E^∗^*.

Together, our results show that these three different regulatory mechanisms produced shifts in smFRET consistent with their predicted effects on channel activity, and suggest that related structural changes occur when the channel shifts between the functionally open and closed states. Previous macroscopic FRET studies of the TM2 reporter site (R151C) also reported a shift upon inhibition by PIP_2_ (27), but suggested that the distance decreased at this site upon channel closure. This difference probably arises from a number of factors as discussed above. In particular, despite the successful use of lifetime measurements to resolve populations with different decay lifetimes, bulk fluorimetry and ensemble measurements are insufficient to distinguish between heterogeneously labeled subpopulations. By contrast, the method we applied here (specifically, ALEX/FAMS) has the ability to sort these individual populations and filter extraneous singly or multiply labeled species. Furthermore, previous studies involving structural modeling of the closed-to-open-state transition in KirBac1.1 were limited by the absence of open-state structures and used a relatively rigid body rotation of the TMs and CTD to produce an open-state model (1, 27). However, more recent crystal structures of both eukaryotic and prokaryotic Kir channels in either open or partially open conformations suggest that these movements are more complex than was previously anticipated.

Our understanding of how a K^+^ channel opens and closes has mostly been derived from structural studies of the prokaryotic KcsA channel, where the physical gate is formed at the intersection of the pore-lining helices and channel opening involves a bending of TM2 to allow opening of the pore (3, 28). Therefore, a simple pore-dilation model might indeed suggest that the R151 sites would move closer together upon channel closure and vice versa. However, in KirBac1.1, optimal packing of the TM helices in the closed state produces a physical gate at F146 that is located above the R151C reporter site (Fig. 4). Additionally, studies of the related KirBac3.1 channel suggest that channel opening involves both bending and rotation of TM2 along with a twisting of the CTD (3, 4, 5). Consequently, if the TM helices twist and rotate to shrink the pore at F146, then geometric considerations would predict that the R151 sites (i.e., below F146) would actually move apart upon channel closure, not get closer together (29). This particular twist-to-shrink model relies on the optimal packing restrictions imposed upon the helices by the membrane environment and is illustrated in Fig. 4. Importantly, the predictions made by this model are consistent with our experimental observations of a decrease in *E^∗^* for this reporter site upon channel inhibition (i.e., closure) by H^+^ and PIP_2_, and an increase in *E^∗^* upon a shift in the gating equilibrium in the opposite direction with the activatory V145L mutation.

Structural movements within the CTD are more difficult to predict because relatively few differences are seen in the CTD for the open- and closed-state structures that have been solved so far; only rigid body rotations relative to the TM helices have been observed (3, 4). Nevertheless, this result clearly demonstrates that the distance between G249 residues within the CTD changes during channel gating, and this structural constraint will be useful for the interpretation of other gating models (3, 4, 5, 6, 24, 26). The Δ*E* shifts reported here represent time-averaged FRET data for individual stochastically labeled molecules because the diffusion time for molecules through the confocal volume (∼1 ms) is relatively slow in comparison with the conformational changes that occur during channel gating. However, in the future, it should also be possible to use smFRET to determine real-time conformational changes that occur during this diffusion time, thereby enhancing the utility of this approach.

In summary, this study demonstrates that solution-based smFRET can now be used to study gating in an ion channel assembled from multiple identical subunits. Critical to this process was the incorporation of the KirBac channels into nanodiscs, which facilitate this solution-based approach yet retain the channels in a bilayer-like environment. This study also highlights how large numbers of single ion channel molecules in a lipid bilayer can be imaged without the requirement for wide-field imaging/scanning or particle tracking, and illustrates that this solution-based approach is not limited to soluble monomeric proteins. We have also shown that this method can detect subtle structural changes in the HBC gate of a K^+^ channel that support the twist-to-shrink model of pore contraction and dilation. With further developments, this single-molecule technique has the potential to record real-time changes in smFRET. Such information could provide a more dynamic picture of gating in multimeric ion channels and allow a detailed quantification of intraprotein distance changes that could then be used to apply structural constraints to different models of channel gating.

## Author Contributions

All experimental work was performed by E.E.S. The study was conceived and supervised by S.J.T. and A.N.K. All of the authors contributed to analyzing the results and writing the manuscript.

## Supporting Material

Document S1. Supporting Materials and Methods and Figs. S1 and S2

Document S2. Article plus Supporting Material
