# Supplementary material for: Solution-Based Single-Molecule FRET Studies of K+ Channel Gating in a Lipid Bilayer
Source: Biophys J. 2016 Jun 21;110(12):2663–70. doi: 10.1016/j.bpj.2016.05.020 (PMC4919593; doi:10.1016/j.bpj.2016.05.020)
Supplement: Document S1. Supporting Materials and Methods and Figs. S1 and S2 [file mmc1.pdf]

Biophysical Journal, Volume *110*

Supplemental Information

Solution-Based Single-Molecule FRET Studies of K^+ Channel Gating in a Lipid Bilayer

Emma E. Sadler, Achillefs N. Kapanidis, and Stephen J. Tucker

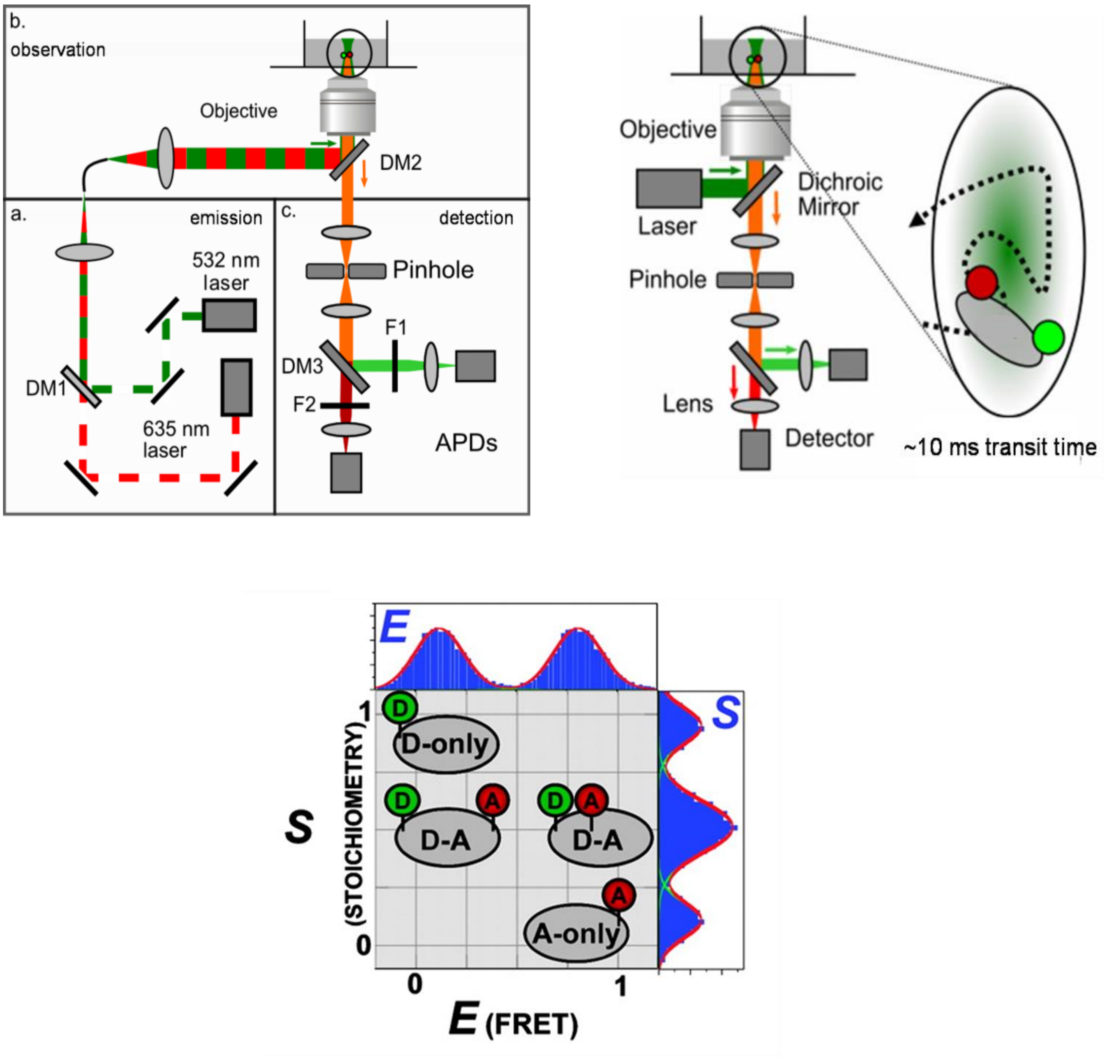

Supplementary Figure S1: **Top Left Panel**: schematic representation of confocal microscopy with Alternating-Laser Excitation (ALEX) used in this study. The beam paths are combined using the dichroic mirror DM1, and directed to the objective, where freely diffusing molecules pass through a femtoliter volume defined by the focused laser beams and confocal optics. The fluorophores are excited by the laser light and the resulting signal from fluorescent decay and emission is directed to further beam splitters (the dichroic mirrors DM2 and DM3) and detected by avalanche photodiodes (APDs) after further filtering extraneous wavelengths using filters F1 and F2. (a) is the emission sector, (b) the observation sector, and (c) the detection sector. **Top Right Panel**: The confocal optics define a femtoliter observation volume. The diffusion constant of the solution is such that only one fluorescently labelled molecule is likely to be traversing the observation volume at any one time. **Bottom Panel**: A stereotypical histogram illustrating the characteristic E^* and S values of D-only, A-only, and D-A doubly-labelled single molecules. This method allows imaging of large numbers of single molecules and sorting of the differently labelled species. The two D-A species have different distances between the donor and acceptor, and thus have different E^*; a higher E^* (FRET) value is characteristic of a shorter D-A separation, *i.e.* more efficient FRET (adapted from reference 4).

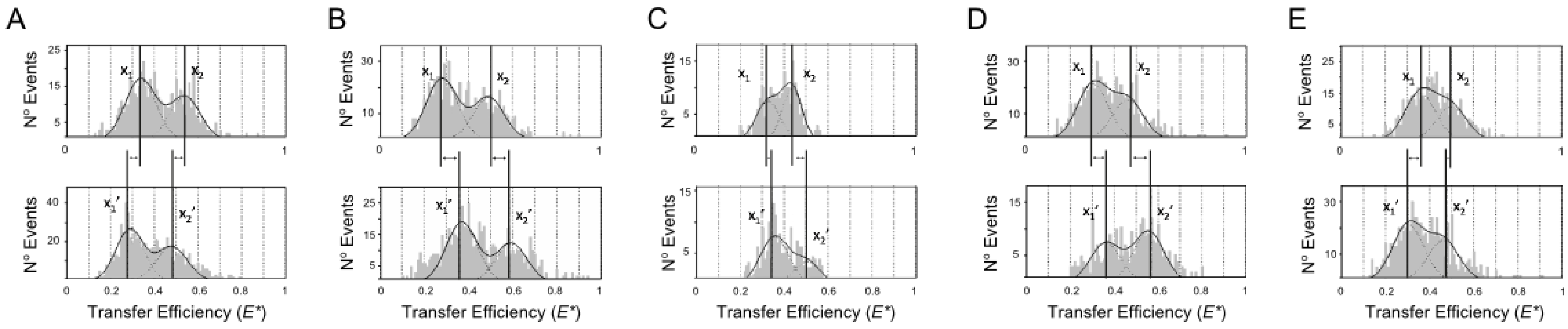

Supplementary Figure S2: Example E^* histograms showing the shift in E^* for: **A:** R151C reporter site upon inhibition by PIP_2. **B**: R151C reporter site upon activation by the V145L mutation. **C**: G249C reporter site upon inhibition by H^+. **D**: G249C reporter site upon inhibition by PIP_2. **E**: R151C reporter site upon activation by the V145L mutation. The vertical bars are used to indicate the relative shift. Data shown are typical of those used to calculate the uncorrected values shown in the table on p4. Note that activation and inhibition produce shifts in opposite directions and that this direction is different between the two reporter sites. These effects are summarised in Figure 3B.

Supplementary Methods

Confocal microscopy

All single-molecule fluorescence experiments were performed at room temperature (18-22˚C) on doubly labelled codon-optimized KirBac1.1 in observation buffer (20 mM HEPES pH 7.5, 150 mM KCl, 0.5 mM EDTA) using confocal-in-solution microscopy with Alternating-Laser Excitation (ALEX) at 10 kHz between 532 nm (120 µW continuous wave (CW), Samba, Cobalt) and 635 nm (30 µW CW, CUBE, Coherent), as described in Santoso *et al.* (1), and illustrated schematically in Figure S1A. The two beam paths from the alternating lasers are combined using a dichroic mirror (DM1) and directed through a series of lenses and pinholes which act to define the observation volume. Aqueous samples are pipetted directly onto a coverslip resting on top of an oil immersion objective (60x, NA=1.35, Olympus UPLSA 60XO) focused to 20 µm above the coverslip surface. Fluorophores attached to freely diffusing molecules are excited as they pass through the observation volume, and the resulting photon streams from fluorescent decay and emission are directed through two beam splitting dichroic mirrors, the first to separate out the longer wavelength fluorescence emission from the excitation laser light (DM2), and the second (DM3) to split the donor and acceptor beams to send them to separate avalanche photodiodes (APDs; SPCM-AQR-14, PerkinElmer). F1 and F2 are filters which exclude extraneous wavelengths, as the dichroics allow passage of a band of frequencies, rather than a single wavelength. Photon arrival times are recorded and analyzed using custom-written software in LabVIEW (National Instruments) and MATLAB (MathWorks, Natick), which detects photon bursts and fits one or more Gaussian distributions to the results (see below). The pinhole of the confocal optics (set to 200 µm), shown in the detection sector (c) of Figure S1A and illustrated in more detail in Figure S1B, defines the observation volume, the size of which is on the order of femtoliters. Taking into account the diffusion constant of the labelled molecules in solution, this means that that there will likely be only one (or zero) fluorescently labelled molecule(s) traversing the observation volume at a given time, making confocal-in-solution microscopy a single-molecule technique.

Alternating-Laser Excitation and Fluorescence-Aided Molecule Sorting

The emission and excitation lasers are both driven by a LabVIEW Virtual Instrument which ensures that data collection is coupled with information about which laser is active at the instant of observation. The laser illumination excites the fluorescent labels, which then decay either directly or via non-radiative transfer to a partner, producing a fluorescence signal which can be detected in either the donor-emission D_{em} or acceptor-emission A_{em} channel, respectively. Alternating-laser excitation (ALEX) produces four photon streams: $F_{Dex,Dem}$, $F_{Dex,Aem}$, $F_{Aex,Dem}$, and $F_{Aex,Aem}$, where $F_{Xex,Yem}$ is the photon count detected in the Y-emission wave length upon excitation with the X-excitation laser. Fluorescence bursts corresponding to molecules detected diffusing through the observation volume are identified using the algorithm described by Kapanidis *et al.* (2) and Lee *et al.* (3). Apparent FRET efficiency (E^*) and Stoichiometry (S) are calculated for each burst using the following equations: $E^* = F_{Dex,Aem}/(F_{Dex,Aem} + F_{Dex,Dem})$ and $S = (F_{Dex,Aem} + F_{Dex,Dem})/(F_{Dex,Aem} + F_{Dex,Dem} + F_{Aex,Aem})$. E^* and S depend on the transfer of fluorescence between the fluorophores, which in turn is highly dependent on the distance between them: $E^* = 1/(1 + (r/R_0)^6)$, where r is the distance between fluorophores, and R_0 is the Förster radius of the dye pair.

The data from the fluorescence bursts are collated into a 2D histogram of FRET efficiency *E** and Stoichiometry *S*, as described in Kapanidis *et al.* (2), and illustrated schematically in Figure S1. It is possible to discern distinct populations of donor-only, acceptor-only, and donor-acceptor labelled molecules using FAMS. As *E** (FRET efficiency) is dependent upon donor-acceptor distance, if there are populations of doubly labelled molecules with different donor-acceptor distances, these will also appear as distinct populations on the histogram: a higher *E** (FRET) value is characteristic of a shorter D-A separation, *i.e.* more efficient FRET, and vice versa.

Corrected and absolute smFRET values

The smFRET values presented are uncorrected values, as opposed to corrected absolute smFRET efficiencies. Cross-talk between the donor and acceptor channels, differences in efficiency of fluorescence transfer and collection efficiency, and differences in quantum yield between the donor and acceptor fluorophores can account for significant discrepancies in absolute values compared to 'raw' values. However, relative efficiencies (*i.e.* shift in *E**), remained unchanged, as can be seen in the table below.

		R151C (TM2)		G249C (CTD)	
		uncorrected ΔE ± std	corrected ΔE ± std	uncorrected ΔE ± std	corrected ΔE ± std
V145L	x_1	0.081 ± 0.011	0.084 ± 0.012	-0.063 ± 0.021	-0.067 ± 0.023
	x_2	0.092 ± 0.024	0.055 ± 0.014	-0.040 ± 0.033	-0.029 ± 0.024
H^+	x_1	-0.063 ± 0.027	-0.070 ± 0.027	0.054 ± 0.021	0.055 ± 0.023
	x_2	-0.082 ± 0.012	-0.053 ± 0.008	0.078 ± 0.023	0.058 ± 0.019
PIP_2	x_1	-0.042 ± 0.009	-0.044 ± 0.009	0.056 ± 0.008	0.058 ± 0.009
	x_2	-0.045 ± 0.011	-0.029 ± 0.007	0.068 ± 0.008	0.045 ± 0.005

These accurate estimates of smFRET efficiencies were obtained by following the approach outlined by Lee *et al.* (3). Using this method to correct the offset seen in Figure 2 we calculate smFRET (*E*) values which correspond well to the actual physical separations (proximal/distal) of R151 and G249 as determined from the crystal structure of KirBac1.1 (PDB: 2WLL) i.e. [16.0 Å, 22.8 Å] and [44.3 Å, 64.2 Å], respectively.

Dye linker modelling

Additionally, modelling of the mean centre position of the dye and its radius of gyration when its non-zero size and linker length is taken into account has been performed using custom-written software (FPSgui). Application of the resulting potential spheres to the PDB files allows calculation of the interdye distances allowing for rotation, and gives values which agree with the corrected smFRET values. However, it should be noted that while the restriction owing to the protein surface can be accounted for, without a model of the bilayer, it is not possible to account for restrictions imposed by the membrane.

Supporting References

1. Santoso, Y., C.M. Joyce, …, A.N. Kapanidis. 2010. Conformational transitions in DNA polymerase I revealed by single-molecule FRET. Proc. Natl. Acad. Sci. USA. 107:715-720.

2. Kapanidis, A.N., N.K. Lee, …, S. Weiss. 2004. Fluorescence-aided molecule sorting: analysis of structure of interactions by alternating-laser excitation of single molecules. Proc. Natl. Acad. Sci. USA. 101:8936-8941.

3. Lee, N.K., A.N. Kapanidis, …, S. Weiss. 2005. Accurate FRET measurements within single diffusing biomolecules using alternating-laser excitation. Biophys. J. 88:2939-2953.
